# Home-Based Intelligent Exercise System for Seniors’ Healthcare: The Example of Golf Croquet

**DOI:** 10.3390/sports11110207

**Published:** 2023-10-26

**Authors:** Yu-Li Zhou, Chia-Li Chen, Shih-Jui Chang, Bo-Sheng Wu

**Affiliations:** 1 Taiwan Economy and Industry Association, Taipei 100008, Taiwan; olivia.chen@hyiasset.com; 2 Department of Information Management, Lunghwa University of Science and Technology, Taoyuan 333326, Taiwan; carey71@gm.lhu.edu.tw (C.-L.C.); qwas90101@gmail.com (B.-S.W.); 3 Center for Professional Competency Certification, Lunghwa University of Science and Technology, Taoyuan 333326, Taiwan

**Keywords:** technology acceptance model, Maslow’s hierarchy of needs, golf croquet, mobile applications, companion robot, AIoT, senior

## Abstract

In 2020, the world experienced the threat of the COVID-19 epidemic, and seniors and chronic disease patients generally reduced their exercise and social activities to avoid increasing the risk of infection, which could lead to increased loneliness and even many diseases. Combining golf croquet games with a mobile application (App) and AIoT companion robots, this research constructs a home-based intelligent exercise system, uses the technology acceptance model (TAM), deduces users’ intention to use this system based on perceived usefulness and perceived ease of use, and adds the needs of love and belonging, esteem, cognitive, aesthetic, and self-actualization in Maslow’s hierarchy of needs theory (HNT) to conduct an analysis of system needs. This study collected empirical data, totaling 320 participants including seniors and their caregivers, from elderly care centers in northern Taiwan using a cross-sectional survey and purposive sampling. Based on regression and variance analysis, the results show that participants have a high level of acceptance of this system, believing that it is easy to learn and operate and can increase interaction with others, improve self-confirmation, satisfy the thirst for knowledge, increase the feeling of happiness, and fulfill self-actualization needs. In the future, by collecting and recording the process of seniors using the App, so as to find out their health problems as soon as possible, expand their daily life through this exercise, and achieve the goal of happy living and better healthcare.

## 1. Introduction

With economic advancement and the progress of science, technology, and medical treatments, human life expectancy is continuing to grow. Indeed, the global aging population is increasing year by year, which has become a topic of close attention by many countries [1,2,3]. With the change in society and the change in the concept of procreation in Taiwan, its population growth has slowed down, and its national fertility rate has decreased year by year. In addition, the prevalence of late marriage and the rise in average life expectancy have significantly changed the age structure of Taiwan’s population [4]. The social problems of aging span not only the needs of the elderly’s welfare, but also economic, medical, and family issues. Secondary issues relate to the elderly’s life, leisure and entertainment, well-being, and psychological and social adaptation, which the government and society should pay attention to and properly plan for [5,6,7].

For strong and rich countries, the greatest risk for seniors is not entirely health problems but also isolation and loneliness [8,9,10]. For seniors, regular exercise is the most effective way to reduce organ degradation and improve physical aging. By participating in leisure sports or activities that they are interested in and obtaining happiness from them, people can achieve physical and mental satisfaction and avoid isolation and loneliness [11,12]. If seniors actively participate in leisure activities that are beneficial to their body and mind and recognize the significance and importance of leisure to quality of life, then they can successfully move toward aging and reduce their social burden [13,14].

Among many sports available to most people, golf croquet is a low-intensity sport and a leisure activity with both fitness and social functions. Seniors typically have plenty of time to participate in croquet, which has a great effect on physical and mental health [15]. In addition to gaining recognition among peers, they can temporarily get rid of unhappiness and become happy from this activity, and depression and anxiety can be effectively relieved. To enable seniors to use it at home, it is necessary to improve the limitations of the open and flat field and the assistance of many staff members needed in the game of golf croquet. The present study develops a golf croquet scoring system to optimize this game and displays and calculates the score and ranking using a mobile application (App). The system combines Internet of Things technology, a pressure sensor, and a Zenbo robot, and its patent was applied for in the Republic of China (Patent No. TWM602930). To explore a sports system suitable for seniors to use at home, this study takes golf croquet as an example and proposes a revised hierarchy of needs theory (HNT) and technology acceptance model (TAM) to confirm seniors’ demand for a healthy home exercise system.

In addition to anticipating the convenience of life and the development of civilization brought by new technology, people may also experience exclusion due to the uncertainty and unknown risks of technological trends. Many scholars have cited the TAM to enhance users’ willingness to use new technologies [16,17,18,19]. As proposed by Davis et al. [20], TAM was designed for users’ behavior in accepting new information systems while analyzing various factors that affect user acceptance. It also provides a theoretical basis for understanding the impact of external factors on the beliefs, attitudes, and intentions of users’ internal factors and then on their use of technology.

People’s desires and impulses are interrelated, and what drives human beings is a number of constant, hereditary, and instinctive needs. These needs are not only physiological but also psychological. They are inherent in human nature, and culture cannot stop them but only restrain them. Scholars have cited HNT to discuss people’s internal needs for things that then influence their behavioral motives [21,22,23,24]. Proposed by Maslow [25], the HNT is a well-known motivational theory and a classification system aimed at reflecting the general needs of society as its foundation and subsequently developing into more acquired emotions. Starting from the lowest level of physiological needs, when one level of needs is met, one will then pursue the higher level of needs of the next level until reaching super self-actualization [26]. 

This study retains perceived usefulness and ease of use in the technology acceptance model and adds Maslow’s demand theory to find out what factors elderly people think will affect their willingness to use a golf croquet exercise system, which can be taken as a reference framework for developing a seniors’ sports management system in the future. An investigation of the golf croquet home exercise system in this study further explores the testers’ acceptance of the functional orientation and demand design of the system. In the future, relevant information during the use process can be collected and recorded, can be used to detect the health problems of users early, and can promote this research to penetrate the daily lives of seniors.

The rest of the paper is structured as follows. Section 2 provides a literature review. Section 3 introduces the methodology used herein, including Maslow’s needs theory to improve the TAM, the questionnaire design, and the analysis methods. Section 4 describes the data collection and analysis of the results, including sample description, reliability and validity analysis, hypothesis verification, and discussion. Section 5 presents the conclusion. Section 6 offers the contribution and applicability of the study, and the limitations are stated in Section 7.

## 2. Literature Review and Research Hypotheses

### 2.1. The Needs and Characteristics of Seniors

Population aging is a global problem, and Taiwan is no exception. Due to the substantial improvement in living standards and the continuous progress of medical technology, science, and technology, people’s awareness of health and wellness is also gradually rising. According to data from a survey report on the elderly by the Ministry of Health and Welfare of Taiwan [27], about 71% of the seniors feel that their health and physical and mental functions are at the middle level—that is, most seniors are still in a relatively healthy state. Therefore, when seniors have relatively more time at their disposal, the arrangement of daily life not only can replace their work style before retirement but also become an important key to maintaining quality of life. In other words, the arrangement of various leisure and health activities is more important for seniors, and this changing trend is also of wide concern to many in academic circles [28,29].

According to data from that same survey report on seniors [27], about 76% of Taiwan’s 55-year-old citizens believe that their physical and mental health is in an ordinary or even good condition. The statistical data also show that “health care group activities” have the highest participation rate, and “leisure and entertainment group activities” are also those they often participate in, regardless of whether the elderly population is aged 55 to 64 or aged 65 or older. This means that Taiwan’s seniors have a strong demand for leisure and healthcare activities. On the other hand, the proportion of seniors over 65 who need nursing, maintenance, or care services is only 17%. Although the physiological function of elderly people will inevitably decline, their psychological pursuit of respect, attention, and companionship will not disappear with age but will continue to increase [30]. With the progress of medical treatment and technology, the maintenance of elderly people’s physiological functions is also improving. It is expected that the number of elderly people over 80 years old in Taiwan’s elderly population will continue to increase.

### 2.2. Recreational Sport and Golf Croquet

Leisure comes from the Latin word “Licere”, which means free time or free activities after getting rid of productive labor [31]. Leisure sports refer to those in which participants freely choose to engage for their own fun during leisure time. Such sports include physical exercise and recreational sports [32,33]. Leisure sports emphasize personal will and self-development, the pursuit of health and physical development, and achieving the purpose of relaxation. More importantly, one can obtain internal satisfaction from sports [34].

With the transformation of society, machines are replacing human beings in various tasks, the number of working hours of human beings has gradually decreased, and the amount of free time has increased. Therefore, how to arrange appropriate rest and life outside work has increasingly attracted public attention. After entering the elderly age group, individuals mainly participate in physical leisure activities (such as aerobics or walking), intellectual leisure activities (such as chess), religious leisure activities (such as charitable activities), and social activities (such as volunteering). If the number of leisure, recreational, educational, and sports courses can be increased and some opportunities can be provided to participate in voluntary services for seniors, then this will fill their spare time and give them the opportunity to participate in society. The most important thing is to help them establish a correct self-awareness so that they will not think that they have lost their usefulness in society [35].

Activity or exercise forms a health guide designed for the human body. If an individual does not engage in a certain degree of activity or exercise, then muscle strength will gradually decline with both age and the aging phenomenon, resulting in a drop in activity, which will increase the possibility of accidents in seniors and relatively increase the risk of chronic diseases [36]. Croquet is very suitable for seniors to participate in and reduce various chronic diseases [15,37]. For them, participation in croquet can help develop interpersonal relationships and interactions after retirement. It is a low-intensity sport, has fitness and social functions, and is quite suitable for the elderly. By participating in croquet, seniors can gain the recognition of their peers and temporarily get rid of unhappiness with this game. Depression and anxiety can thus be effectively relieved [35,38].

Croquet originated in France. It is a recreational activity that can be participated in by many people, and it can help with mind power [39]. However, it is also subject to some restrictions, including limitations in field size. Golf croquet originated in Japan and drew lessons from the traditional British croquet sport that rose in the 14th century. At that time, it provided children with a means of entertainment, but it was not accepted by them; instead, it was loved by seniors. It is an indoor sport aptly suitable for seniors [40,41].

**Figure 1:**
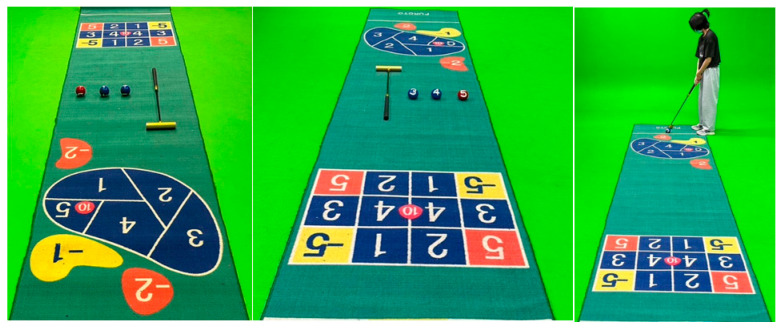
Schematic diagram of golf croquet.

Golf croquet combines fun and brainstorming. It integrates the training of feet, hands, and concentration into an activity so that participants can also stand around, chat, and walk happily and naturally. Golf croquet requires more attention and better control, and the rich colors on the scoring carpet improve the willingness to participate. The score matrix in Figure 1 is 13 grids, indicating different scores. The score is doubled when the ball falls at the selected number. Scores can also be negative. The arrangement of competition activities can make everyone more aware of the importance of cooperation and fully open the door to group activities where people are able to laugh and enjoy time with others.

### 2.3. Internet of Things and Companion Robot

Taiwan’s society is facing the phenomenon of aging and fewer children, causing a hidden worry about the country’s sustainable development. How to guarantee the quality of life of seniors and reduce the burden of young people are important issues for society.

From the perspectives of science and technology, the maturity of IoT technology, and the rapid development of AI, the use of AIoT emerging technology combines AI and IoT to improve the quality of life of seniors and has attracted greater attention [42,43,44,45,46]. IoT means that electronic devices can be connected to the Internet through network infrastructure [47]. The device has a variety of sensors, which can collect and integrate information anytime and anywhere to assist people’s lives [48,49]. In recent years, IoT has been used more and more widely, covering a variety of different application fields. Among them, Qian et al. [50] used ambient assisted living (AAL) and a healthcare monitor (HM) in combination with AI and IoT to systematically help seniors live a more relaxed and better life in terms of methodology and application scenarios. Abdi et al. [51] found that some emerging technologies (such as AIoT) may solve the elderly’s basic self-care and medical care needs, but there is still a gap in their potential use in some nursing fields. Research results have provided the industry with some clear challenges and obstacles to improving the adoption of emerging technologies by the elderly, such as robots and virtual/augmented reality (AR/VR). The integration of IoT and mobile phone devices can effectively solve the difficulties of limited storage space and insufficient computing processing capacity of devices at low power consumption, strengthen the development of the entire IoT technology space, and achieve effective management in terms of reliability, computing performance, security, and confidentiality [43,45,52,53].

With the progress of science and technology, people expect robots to be close to human life and provide more diversified services. Various service robots have begun to flourish, which try to integrate into human life with different roles. In elderly care, the development of many machine pets and machine dolls, which can interact with the elderly, really helps ease their psychological loneliness and lets them feel the value of being needed again [54]. Companion robots have evolved from mental commitment robots to therapeutic robots that provide psychological and spiritual healing effects for the elderly, relieve their stress and loneliness, offer psychological healing functions for patients with dementia, and improve their mental health and quality of life [55]. Studies [56,57,58,59,60] also showed that if you want to improve the quality of life of seniors, then you can use home-based robots to achieve the goal and also effectively reduce the burden of caregivers.

**Figure 2:**
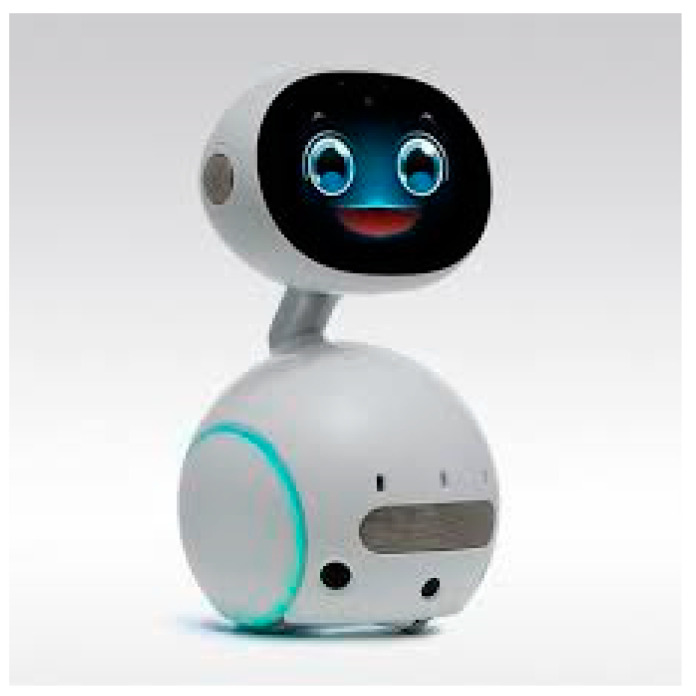
Zenbo robot.

The rise of AI in recent years, especially natural language processing technology, has gradually matured and become widespread. Voice assistants combined with IoT technology have already entered many homes. Users can use their voice to perform functional tasks or information queries through a voice assistant, which can even be applied to the biomedical field, including disease diagnosis and life assistance [61]. Many research and development projects also try to combine robots and natural language processing to develop robots that can talk naturally. For example, Zenbo (as shown in Figure 2), a robot developed by ASUS, is a companion robot that combines the cloud natural language processing engine. Our work uses ASUS Zenbo to connect to our App. Zenbo Software Development Kit and additional tools can accelerate our development of other Apps and capabilities for Zenbo.

Because Zenbo has a variety of functions, it can cover the basic needs of seniors in daily life and company [62]. Compared with general robots, Zenbo’s price is more reasonable, which increases its popularity for general families or nursing institutions. Therefore, this study uses Zenbo as an auxiliary research tool to deeply explore the application of companion robots and demand analysis of an elderly family home motion system.

### 2.4. Mobile Application and Scoring System

The vigorous development of smartphones and wireless networks allows users to easily access the information they want and need through mobile technology anytime and anywhere. It has created diversified mobile application systems, changed the way people communicate, and improved social interactions. Therefore, more middle-aged and seniors are using mobile phones to take, upload, and share photos or use social software to communicate with friends and relatives. They are enjoying the convenience brought by information technology. According to the Ofcom [63] report on adults’ multimedia use and attitudes, 28% of the elderly aged 75 or above used tablet computers in 2018, or an increase of 15% over 2015. In 2020, 77% of the elderly over 65 used the Internet at home. According to the same survey results, the Internet use rate of the age group 65 to 74 increased from 52% in 2011 to 83% in 2019. This means that seniors are narrowing the generation gap in technology use, and they are not only using their computers but also expanding to mobile phones and tablets [64].

In the “Demand and Impact of the Elderly Consumers” report for the middle-aged and the elderly, AT Kearney [65] pointed out that the elderly have high acceptance of mobile technology, 69% of seniors use the Internet and mobile phones at the same time, and mobile technology has brought changes and influence to the lives of seniors. A report on the use of smartphones published by InsightXplorer [66] explained the use of applications and browsers/web pages on smartphones. Regardless of the content type, the overall use rate of Apps is higher than that of browsers/web pages, and the availability of local services and specific information is higher than that of browsers. According to Dennison et al. [67], mobile phones have become an important way to spread healthy behaviors. The development of Internet mobile technology has promoted a rapid rise in fitness Apps, which are third-party application(s) of smartphones or wearable devices that can help users record fitness data, guide sports learning, and lead healthy lifestyles [68]. Thus, more and more scholars are continuously investing in research related to fitness or sports applications [69,70,71,72]. Under such consideration, people can effectively and immediately manage their own health conditions and make health management plans using mobile Apps.

Most intelligent sports systems are mainly targeted at young people, and their category is also biased toward running or cycling records and fitness teaching. This may motivate people to try to mimic observed activity patterns, resulting in more or fewer sports activities depending on the sports behavior of their peers. Products similar to this research include the golf practice system, which focuses on an evaluation of swing strength and golf trajectory [73,74]. Clubs on Strava [75] form the largest online social network in the field, connecting millions of runners, cyclists, hikers, walkers, and other active people. Franken et al. [76] found Clubs on Strava influence people’s running behavior. A sports partner’s excellent results will motivate another follower or friend to keep exercising [75]. There are few indoor sports intelligence systems for multi-person interactive seniors, so it is very important to discuss multi-person online sports systems suitable for seniors.

Based on the above, we find that using mobile Apps to promote health management products is very effective. With the prototype development of a home-based intelligent exercise system, this research can attract Taiwanese to pay attention to their own health, immediately know health management suggestions, and use this App to achieve the purpose of health maintenance. In order to adapt to the sports system used by seniors at home, this study takes golf croquet as an example to improve the croquet field and rule restrictions, based on the research result from “Auto scoring system for golf strike back ball” published by Chen et al. [77]. 

**Figure 3:**
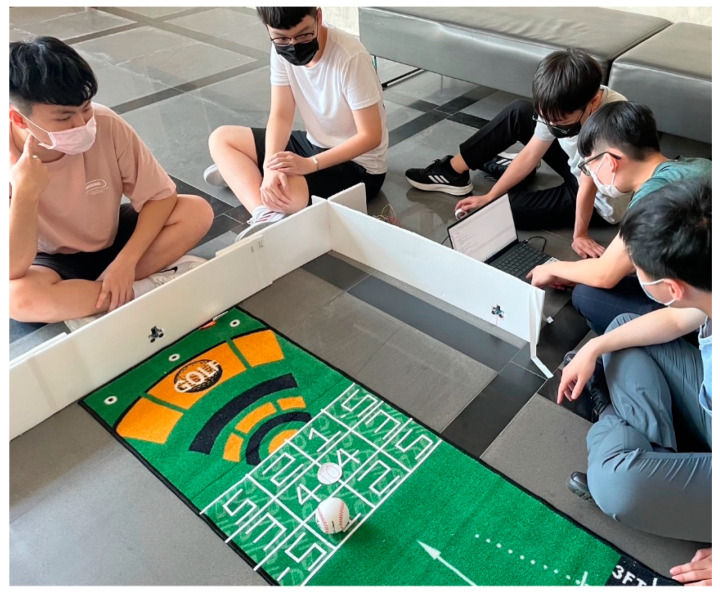
Ultrasonic sensors plate and image recognition device.

**Figure 4:**
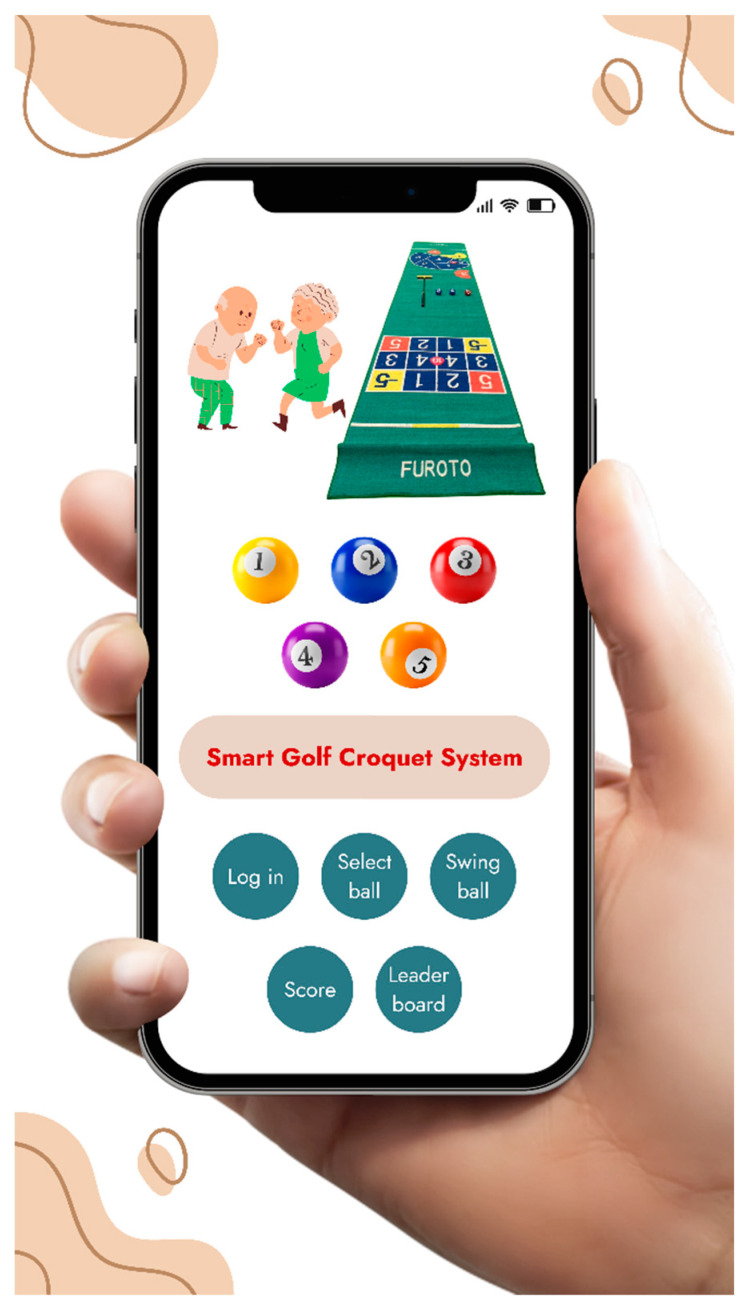
An App for the auto scoring system for golf strike back ball.

This system is part of a new patent application (No.: TWM602930). As the basis for the score of golf croquet, it uses a sensing plate and image recognition device (as shown in Figure 3) to determine the path and final stop position of the croquet after hitting. The score is recorded and displayed in the App software. The “Smart Golf Croquet System” App in Figure 4 includes five functions: login, select ball, swing ball, score, and leader board. It also can automatically sort the scores and list the rankings, so as to reduce the number of judges, scorers, and other staff. A smaller venue can also be used. This system improves the original restrictions and also records the muscle groups and fitness conditions used by users in the process of use because the App software of this system contains an image recognition function module and storage area that can identify and store at least three hitting paths. Thus, the base point of hitting can be set in advance with assistive devices. Rehabilitation therapists suggest that three hitting points be determined for fixed muscles. The hitting path, the position of the ball, and scoring are recorded at the same time, and so specific muscle rehabilitation or fitness status can be evaluated.

When playing traditional golf croquet, there will often be an embarrassing situation where the ball is pressing on the line or many points. At this time, players will be confused about the score. Our work changes it to an intelligent scoring method, using image recognition to detect the actual score of the ball, sending it back to the background to calculate the score, and then making a sound to inform the score. This is convenient to help users easily know the score overview. The intelligent scoring system mainly uses the camera that shoots the scoreboard, backend differential image recognition, and finally, the score read-out. 

In smart golf croquet, there are four main items: smart scoring system, smart club, robot, and App. The intelligent scoring system mainly uses the camera that shoots the scoreboard, then loads the differential image recognition at the backend, and finally reads the score out loud. The smart club will have installed a shock, pressure, and accelerometer sensor, and an Arduino processor will process the data. The robot has an intelligent voice and camera system. Usually, the elderly is not an ideal age group for using electronic products and can operate this system only by speaking. Furthermore, the App screen can be directly displayed on the robot’s screen. The last part of the App integrates the data of previous scoring and clubs for display. Next, multiplayer connection is the most important part, so that players can play together online without being disturbed by space factors.

### 2.5. TAM and Hypotheses

TAM, published by Davis et al. [20], is based on the theory of rational behavior. Davis believed that an individual’s attitude will affect his/her willingness to use something and then affect his/her actual behavior. They proposed that “perceived usefulness” and “perceived ease of use” are the key factors that affect his/her acceptance or not and added external variables, including system design characteristics, user characteristics, use environment, task characteristics, degree of involvement, etc., to explore their relevance with a user’s cognition and behavioral intention. In other words, because of the influence of some external factors, users realize whether the new technology is useful and easy to use, and only after they have a positive attitude toward the internal psychology, will they have the intention to use, and then the actual use behavior will occur.

The TAM dimensions defined by Davis et al. [20] are as follows. (1) Perceived usefulness: external variables and perceived ease of use affect perceived usefulness, improvements in the ease of using the system promote work efficiency, and perceived usefulness affects people’s attitude and intention to use new technology systems. (2) Perceived ease of use: perceived ease of use affects perceived usefulness and use attitude because users need to be able to use it to produce performance. The easier the technology system is to use, the better the work efficiency will be, and the time to complete the task will be shortened. (3) Intention to use: perceived ease of use and attitude to use affect people’s intention to use the technology system. We expect that the technology system can improve work performance and feelings about the system and encourage users to actually use the product. Research on the acceptability of interpreting information systems based on TAM has obtained much empirical evidence [78,79], which has been fully validated in terms of both interpretation ability and theoretical applicability. Although TAM was originally only used in information-related fields, many studies have applied it to information services and innovation and technology products [80,81], proved its effectiveness, and verified the relationships among users’ perceived usefulness, perceived ease of use, use attitude, intention to use, user behavior, and other aspects of information technology.

#### 2.5.1. Mobile Phone Experience and Education Level

Experience and education level are decisive factors of intention to adopt, which directly leads to people’s willingness to use home-based intelligent exercise systems. Experience indicates the benefits that customers can obtain when using home-based intelligent exercise systems, which can be a judgment of the total value of the home intelligent sports system [82]. According to one study, experience and education level significantly affect people’s attitudes toward home-based intelligent exercise systems. Schill et al. [83] pointed out that customers’ experience in using home-based intelligent exercise systems determines their purchase intention. Tichenor et al. [84] noted that parents with higher education levels have higher knowledge and experience in using new scientific and technological information and a higher willingness to adopt electronic children’s books. Youn and Lee [85] suggested that experience may affect perceived usefulness and perceived ease of use when using mobile payment. We thus present the following hypotheses.

**H1.**  *Mobile phone experience has a significantly positive impact on the perceived usefulness of a home-based intelligent exercise system.*

**H2.**  *Education level has a significantly positive impact on the perceived ease of use of a home-based intelligent exercise system.*

#### 2.5.2. Perceived Usefulness and Perceived Ease of Use

People’s attitudes, willingness, and actual use behavior toward the acceptance and use of science and technology are mainly influenced by their perceived usefulness and ease of use of the information system. According to Peng and Yan [86], the perceived usefulness of scientific and technological products directly affects the willingness to use multiple media kiosks (MMKs). Hsu and Chang [87] predicted students’ views on the acceptance and use of Moodle, an open-source e-learning system. The results showed that perceived ease of use and perceived usefulness have a significantly positive impact on the attitude toward using Moodle. Wang et al. [88] discussed how to make artificial intelligence (AI) more effective and profitable in e-commerce and how entrepreneurs can use AI technology to help achieve their business goals. The results of model verification showed that perceived usefulness and perceived ease of use also have a positive impact on use attitude and intention. Based on the above research, we make the following hypotheses.

**H6.**  *Perceived usefulness has a significantly positive impact on the intention to use a home-based intelligent exercise system.*

**H7.**  *Perceived ease of use has a significantly positive impact on the intention to use a home-based intelligent exercise system.*

### 2.6. HNT and Hypotheses

Maslow’s hierarchy of needs theory [25] is the most widely used theory in the study of organizational incentives. It aims to reflect the general needs of society as its foundation and has been developed into more acquired emotions. According to this theory, in order to generate motivation in the next stage, each previous stage must be satisfied by the individual, and the greatest function of the hierarchy of needs theory is that it points out that everyone has needs [89]. This theory is important in explaining personality and motivation. It puts forward that the internal motivation of individual growth is motivation, motivation is composed of various needs of different levels and natures, there are high and low levels and sequences among various needs, and the degree of demand and satisfaction at each level will determine the realm of individual personality development [90].

The theory includes the following requirements. (1) Physiological needs: the basic needs for human survival, including food (hunger, thirst), clothing, housing, transportation, education, and happiness. (2) Safety needs: including physical and psychological safety and stability, avoiding physical injury or mental injury. (3) Social needs: various interpersonal relationships for personal needs, such as friendship, love, companionship, and sense of belonging. (4) Self-esteem needs: personal needs related to self-esteem, such as the pursuit of social status, social respect, recognition, and trust. (5) Cognitive needs: seeking knowledge and the curiosity of individuals to explore specific problems and knowledge and then learn. (6) Aesthetic needs: seeking beauty, including the appreciation of external beauty and the internal desire to achieve perfection in the things within. (7) Self-fulfillment needs: the highest level of demand in this theory refers to the individual’s ability to maximize, realize his/her ideal and ambition, and become his/her desired person. (8) Spiritual needs: cover the needs to transcend oneself and integrate nature and humans, including altruism, compassion, and other goodness implied in human nature.

Maslow’s hierarchy of needs theory is not only a prominent science in psychology but also an important exposition of humanistic psychology and is widely used in other fields. Ryan et al. [91] proposed a framework for community stability and sustainability during COVID-19. Their study pointed to the need for policymakers to understand associated risks and how Maslow’s hierarchy of needs and social determinants of health can guide policy across society. Aligning decision-making with societal needs will help ensure that the needs of all segments of society are met while managing a crisis. Altymurat et al. [92] discussed the application of Maslow’s hierarchy of needs principles in organizations, showing that companies will function optimally when the requirements for confidentiality, convenience, and certainty are met, thereby enabling the satisfaction of the process of user knowledge requirements to go smoothly. Hale et al. [93] proposed Maslow’s hierarchy of human needs (physiological, safety, love/belonging, esteem, and self-actualization) as a potential framework for addressing wellness programs. Their findings revealed that widespread burnout in graduate medical education exists and has detrimental effects on career satisfaction, personal well-being, and patient outcomes.

#### 2.6.1. Education Level

Although it is easy to meet an individual’s low initial social, self-esteem, and self-realization needs, such needs may increase with education level. Orth et al. [94] studied the development of self-esteem from youth to old age. The results indicated that people with higher education have higher self-esteem than those with lower education. Rational users will try to correct this disharmony by distorting or modifying their sense of social existence, emotional belonging, self-expression, and happiness so as to make it more consistent with reality [95,96]. Van Eckert et al. [97] pointed out that the self-esteem level of nurses with high academic qualifications is significantly higher than that of nurses with low academic qualifications, and the relative job satisfaction and high-quality patient care are significant. Therefore, education level will often improve users’ satisfaction with social needs, self-esteem, and self-realization needs, while negation will reduce this satisfaction. Based on the above research, we make the following hypotheses.

**H3.**  *Education level has a significantly positive impact on social needs.*

**H4.**  *Education level has a significantly positive impact on esteem needs.*

**H5.**  *Education level has a significantly positive impact on self-actualization needs.*

#### 2.6.2. Social Needs

Emotional belonging refers to intimate feelings and emotional contact between individuals, including contact intensity and moral support [98]. Sinclair and Dowdy [99] noted that emotional belonging will promote individuals to perceive intimacy in the group and establish better interpersonal relationships with others. Ridings and Gefen [100] stated that the most influential motivation to join a virtual community is to seek social support and friendship. Rau et al. [101] believed that the main motivation for people to participate in social networks is to meet their emotional needs rather than information needs. Therefore, we believe that the satisfaction of emotional belonging significantly affects users’ attitudes toward a home intelligent sports system and then affects their willingness to continue to use it. Based on the above research, we present the following hypothesis.

**H8.**  *Social needs have a significantly positive impact on the intention to use a home-based intelligent exercise system.*

#### 2.6.3. Esteem Needs

Cui et al. [21] confirmed that self-esteem significantly affects the purchase intention of electric vehicles. Lu et al. [102] also supported this hypothesis by investigating wireless Internet service products. In addition, Bi and Zhang [103] proposed a relatively new interactive marketing model: influencer marketing, and the research results showed that the level of self-esteem significantly affects the purchase of endorsed products. People with high status are also prone to accept environment-friendly products to enhance themselves [104]. However, most studies have only demonstrated that self-esteem may affect product adoption intention, neglecting how self-esteem impacts the adoption of intelligent products. Therefore, we propose the following hypothesis.

**H9.**  *Esteem needs have a significantly positive impact on the intention to use a home-based intelligent exercise system.*

#### 2.6.4. Cognitive Needs

Human beings have the need to increase their intelligence and pursue knowledge. Cognitive needs (CNs) form the expression of humans’ natural need for learning, exploring, discovering, and creating so as to better understand the world. If this demand for self-realization and learning growth is not met, then it will lead to confusion and an identity crisis, which directly relates to the need for exploration or the openness of experience [25]. Lin et al. [105] used cognitive needs as a regulator to explore the influence of online comments on purchase intention and found that shoppers with high cognitive needs have a positive impact on purchase intention. Hussain and Shabir [106] pointed out that information professionals need to re-examine the use of social media to meet their cognitive needs because the satisfaction they acquire differs from the satisfaction they seek from social media. Zhao and Zhu [107] discussed the role of cognitive needs in regulating internal mechanisms, and their results showed that cognitive needs, as a regulating variable, significantly affect the influence path from perceived usefulness to attitude and purchase intention. At present, there is no research on testing home-based intelligent exercise systems with the CN variable and linking it with TAM. Thus, we put forward a hypothesis.

**H10.**  *Cognitive needs have a significantly positive impact on the intention to use a home-based intelligent exercise system.*

#### 2.6.5. Aesthetic Needs

Aesthetics needs (ANs) are a higher level of Maslow’s needs. With the development of the economy, most people can meet the basic level of Maslow’s needs, and visual aesthetics (VAs) become more important. As far as a company is concerned, VAs are a symbol that distinguishes it from other competitors. The best example is Apple, which is a powerful measure for the company to compete for increasing customers [108]. According to studies [109,110,111,112], the fact that ANs significantly affect customers’ willingness to use has been proved. However, at present, there is no research on testing a home intelligent sports system with the AN variable and linking it with TAM. Thus, we put forward another hypothesis.

**H11.**  *Aesthetic needs have a significantly positive impact on the intention to use a home-based intelligent exercise system.*

#### 2.6.6. Self-Actualization Needs

The satisfaction of self-realization needs includes the satisfaction of self-expression and the satisfaction of happiness. Self-expression is defined as an individual’s desire to show himself/herself [113]. Daimi and Tolunay [114] researched social media influencers and their influence on consumers’ purchase decisions, noting that the authenticity and credibility of influencers and the self-demand of followers significantly affect their purchase intentions. In addition, both eudaimonia and hedonia relate to life satisfaction and positive emotion [115]. Therefore, when users think that a higher level of self-expression satisfaction can enhance social attraction, they will attain psychological satisfaction, which will further enhance their sustained willingness. Based on the above research, we propose the following hypothesis.

**H12.**  *Self-actualization needs have a significantly positive impact on the intention to use a home-based intelligent exercise system.*

To sum up the above, this study is based on perceived usefulness and perceived ease of use in TAM to explore the relationships among users’ cognition, attitude, intention, and use of technology systems. Next, we explore the needs of seniors, propose to add Maslow’s needs theory to improve TAM, and confirm the relationships among social needs, self-esteem needs, cognitive needs, aesthetic needs, and self-actualization needs in Maslow’s needs theory, as well as perceived usefulness, perceived ease of use, and intention to use of TAM.

## 3. Research Method and Design

**Figure 5:**
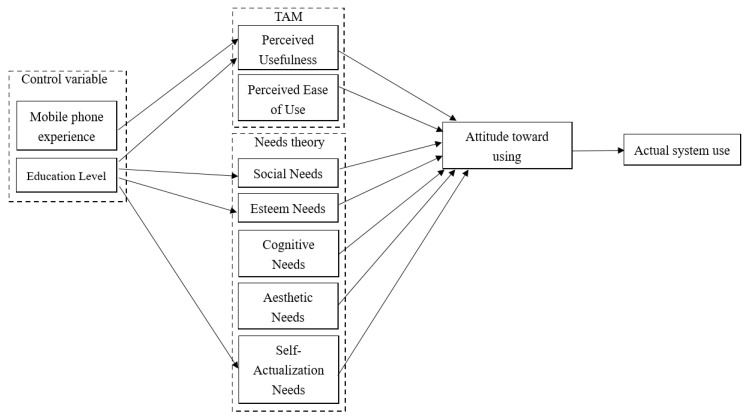
Conceptual framework.

This study proposes to add Maslow’s hierarchy of needs theory to improve TAM and explores the needs of seniors for healthy sports mobile phone apps. It includes the relationship between TAM of perceived usefulness, the perceived ease of use, and Maslow’s needs theory of social needs, esteem needs, cognitive needs, aesthetic needs, and self-actualization needs. The control variables include the relationship between mobile phone use experience and perceived usefulness and the relationship between education level and perceived usefulness, social needs, esteem needs, and self-actualization needs. This study establishes hypotheses by exploring the relationship between these variables and the home-based intelligent exercise system of golf croquet and carries out experimental analysis using a questionnaire. The research structure is shown in Figure 5.

According to the literature, perceived usefulness, perceived ease of use, and intention to use TAM are sorted into 7 criteria and implications and 16 items. Among them, Davis and Bagozzi [20] predicted people’s acceptance of computers by measuring their intentions and their ability to explain their intentions based on their attitudes, subjective norms, perceived usefulness, perceived ease of use, and related variables, thereby improving organizational performance. Venkatesh and Davis [37] developed and tested a theoretical extension of TAM, explaining the perceived usefulness and use intention of social influence and cognitive tool process. The results showed that both the social influence process and cognitive tool process significantly affect user acceptance. Huang [116] applied TAM to explore the use of a tele-healthcare system by community residents, as well as the acceptance of nursing technology products. Kuo [117] used TAM II to explore the influence of subjective norms, impressions, mission relevance, output quality, and results explicability on consumers’ cognitive usefulness of electric vehicles and to explore the influence of cognitive usefulness and cognitive usability conditions on consumers’ intention to use electric vehicles. The application of TAM by Tang [118] aimed to explore the actual use of digital learning by indigenous adults and their acceptance of technology. 

The above criteria and their implications are as follows. (1) Relieve loneliness: can effectively alleviate loneliness. (2) Stay healthy: can maintain physical and mental health. (3) Convenience: very convenient to use. (4) Easy to operate: operation steps are simple. (5) Easy to learn: does not take much time to learn. (6) Self-intention to use: facilitates users to refer to the product and use it in practice. (7) Recommend and share: recommend and share the product with others.

According to the literature, the five items in Maslow’s hierarchy of needs theory are sorted out into 15 criteria and implications and 15 items. Among them, Hsu [119] aimed to understand the importance and satisfaction of middle-aged and elderly people at the level of needs and to explore the distribution of Maslow’s five major needs by using the analysis of importance and performance (IPA). The research results showed middle-aged and elderly people with the ability to prepare in advance and be healthy and active in aging in the future. Chen [120] observed the phenomenon and significance of rural religious belief behavior from tradition to the present with Maslow’s demand theory and discussed the feasibility of developing rural religious creation and activities with the concept of leisure. That study also put forward suggestions for practical development according to the research results.

The above criteria and their implications are as follows. (1) Sense of identity: can identify with each other. (2) Maintain good relationships: can maintain good relationships with others and maintain emotions. (3) Embrace other people: able to accept others and live in harmony. (4) Sense of glory: can exhibit a sense of pride. (5) Earn recognition: able to gain the affirmation of others. (6) Respected by others: be respected by others. (7) Full of confidence: can make oneself full of confidence. (8) Curious and seek knowledge: be curious about new sports equipment and want to know. (9) Learning their skills: easy to learn and want to learn skills for use. (10) Hone their skills: want to use technology frequently to improve skills. (11) System functions: functional design meets the requirements. (12) Font display: the font and screen display are clear and easy to recognize. (13) Exert personal potential: be able to fully exert one’s potential without being disturbed by emotion or environment. (14) Competition with myself: actively face challenges and do not give up easily. (15) Peak experience: experience a high degree of self-realization and feel happy, excited, and touched in a short period of time, even selfless feelings.

All items are measured using the Likert five-point scale, which is divided into five options according to the degree of agreement: very disagree (1 point), disagree (2 points), ordinary (3 points), agree (4 points), and very agree (5 points), as shown in Table 1. In this paper, the selected questionnaire items in Appendix A.

During the literature review, 31 questions in total were collected from a questionnaire consisting of 7 criteria for TAM and 15 criteria for Maslow’s hierarchy of needs theory. First, a sample structure analysis is conducted on the collected questionnaire, and narrative statistics are analyzed and explained for each aspect. Second, confirmatory factor analysis (CFA) is conducted to confirm the results after clustering. CFA is a statistical method used in social sciences to evaluate the degree of fit between theoretical models and real-life collected datasets. It is often used as a construct validity test scale or measurement tool. In other words, its purpose is to test the relationship between observed indicators and potential variables (factors) in the model [121]. Third, regression analysis is conducted to validate the proposed hypothesis. Regression analysis is the most basic and important statistical analysis technique and hypothesis validation method in social science research methods, which is used to display the relationship between two or more variables [122,123]. Finally, an analysis of variance is conducted on the dimensions proposed in this study based on education level and mobile phone usage experience. Analysis of variance helps identify whether differences between data groups are statistically significant. Its principle is to analyze the level of differences within each group by selecting samples from each group [124].

This is a non-interventional study (e.g., surveys, questionnaires, social media research). The process was anonymous and all participants were fully informed as to why the study was being conducted, how the data would be used, and whether there were any associated risks. Based on the regulations announced in Taiwan: Human Subjects Research Act, Chapter 2, Article 5, Announcement of Ministry of Health and Welfare, Executive Yuan, Republic of China (Taiwan) Issue Date: 5 July 2012, Issue No.: 1010265075, this study was conducted under the conditions of ‘Exempt from being submitted to the Ethics Review Committee for review’, please refer to Appendix B for detailed regulations and ethical statements.

This study collected empirical data on subjects from two elderly care centers in northern Taiwan using a cross-sectional survey and purposive sampling. The main subjects are seniors over 65 years old and their accompanying caregivers. The unit under test is a long-term partner of this study. Every semester, students act as volunteers to serve and visit the elderly residents, providing timely physical and mental comfort and support to them, bringing adjustments, leisure, and entertainment to their lives, and thereby improving their quality of life. The quality of life of the residents can be improved by providing timely relief and comfort for the residents’ illnesses and leveraging the power of social resources to achieve a community care model and social interaction.

In order to carry out this research smoothly, before the test, a discussion was held with the person in charge of the two elderly care centers about the relevant content, including the location of the meeting room, space capacity, related equipment, the location of golf croquet, and the size of the venue. The person in charge was asked to communicate to the elderly and accompanying caregivers who can participate in this activity and then compile the number of people willing to participate. In order to carry out this activity smoothly, in addition to stipulating that elderly people who were willing to participate needed to be accompanied by a companion or caregiver who can assist them, in order to maintain a safe distance and avoid contracting COVID-19, the number of people per day/session was limited to 32 people (16 groups, 2 people in each group). The relevant number of participants and the test schedule are shown in Table 2. The total number of participants in elderly care center (A) is 192, and the total number of participants in elderly care center (B) is 128. Due to the large number of people in elderly care center (A), the testing dates were held every Tuesday and Thursday for three weeks, while those for elderly care center (B) were scheduled every Wednesday for four weeks.

Our work uses a physical questionnaire. Before filling out the questionnaire, the individual seniors and their companion or caregivers gathered in a conference room and were verbally explained the rules and disadvantages of traditional golf croquet. The improvement methods proposed in this study were introduced. At the same time, recorded explanations and introduction videos were played to make the participants more aware of the overall testing procedure. After completing the explanation, they were moved to a spacious indoor space to experience the golf croquet home-based intelligent exercise system. Finally, they gradually filled out the questionnaire content based on their experience results. For those who did not understand the questionnaire content, we provided assistance and explained the content of the questions.

The greater the number of samples that are selected, the higher is the accuracy of inferring the actual situation of the statistical analysis results, which can prevent differences from being effectively tested due to too few samples and can reduce the probability of type II errors. However, when the number of samples is too large, subtle differences may cause the research results to reach significant results, increasing the chance of type I errors [125]. The number of samples selected should be adjusted according to the needs of the statistical analysis used in this study. It is best to have more than 300 samples for factor analysis [126], or the number of effective samples should be at least 5 times the number of microscale questions [127]. In addition, depending on the number of questions in the questionnaire, the sample size in this study should be 3 to 5 times or 5 to 10 times the number of questions in the largest subscale [128,129]. The number of questionnaires is 320. The questionnaires were completely recovered, and so the effective recovery rate is 100%, thus presenting sufficient sample representativeness. The following sections describe the data collection and analysis of the results.

## 4. Data Collection and Analysis of Results

### 4.1. Sample Description

The basic data of the collected questionnaire samples are as follows: 82.2% of the respondents are male, 78.4% of the respondents have a high school education, and 80.6% have more than five years of experience in using smartphones. Among the subjects, 31 have chronic diseases, with cardiovascular diseases accounting for the majority (52%), followed by diabetes (26%), and 61% have had chronic diseases for more than five years. Those who participate in sports three times a week account for 49.7% of the sample, followed by more than five times a week at 26.3%. A duration of each exercise of less than 30 min accounts for the majority (74.4%), followed by 1 h (19.4%). The majority (37.2%) of them have continued to exercise for more than five years. The basic information collected with the questionnaire is shown in Table 3.

According to the statistical results in Table 4, the average number of questions on perceived usefulness, perceived ease of use, intention to use, social needs, self-esteem needs, cognitive needs, aesthetic needs, and self-actualization needs of the respondents ranges from 3.97 to 4.13. This represents the respondents’ positive attitude toward the home action system of golf croquet.

### 4.2. Factor Analysis, Reliability, and Validity Analysis

To further understand the degree of consent of the subjects to each dimension, this study carries out factor analysis, respectively, for TAM’s perceived usefulness, perceived ease of use, and intention to use as well as Maslow’s social needs, self-esteem needs, cognitive needs, aesthetic needs, and self-actualization needs. It extracts common factors with a characteristic value greater than 1 [130] using principal component analysis and the maximum variation pivot method. Questions with an absolute value of the factor load greater than 0.5 are reserved and properly classified. In terms of scale reliability, according to Guilford [131], Cronbach’s α greater than 0.7 means high reliability and a value less than 0.35 shows low reliability, which should be rejected [132]. The construction validity of this study is based on the item-total correlation method of Kerlinger [133]—that is, assuming that the total score is valid, the size of the correlation coefficient between individual items and the total score is the measure of construct validity.

The results in Table 5 show that the Kaiser–Meyer–Olkin (KMO) value of each facet is between 0.500 and 0.851. KMO is an index used to compare simple correlation coefficients and partial correlation coefficients between variables. The closer its value is to 1, the stronger the correlation between variables, and the more suitable the original variables are for factor analysis. On the contrary, the closer it is to 0, the weaker the correlation between variables, and the less suitable it is [134,135]. Bartlett’s ball test is significantly in line with the requirements, and the values of each facet are quite good. 

The results in Table 6 show that the factor load of each item is between 0.502 and 0.877, which is more than 0.5. The characteristic value of each facet is between 1.746 and 4.711, the cumulative explanatory variance is between 57.773 and 87.302, and the item-total correlation value of each item is greater than 0.5, indicating considerable constructive validity and content validity. Cronbach’s α values are all greater than 0.6, denoting good internal reliability. There is thus real correlation between the measurement items, and the content of the questionnaire is highly consistent.

### 4.3. Hypothesis Testing

Table 7 shows that, to predict the intention of use, each dimension uses SPSS software to carry out regression analysis. According to the research framework (Figure 5), it is assumed that the path direction variable is the dependent variable, and the path starting variable is the independent variable or predictive variable. According to the analysis of the relationship between perceived usefulness and intention to use, the empirical results show that hypothesis H6 is supported, indicating that perceived usefulness has a significantly positive impact (*p* < 0.001) on intention to use. 

According to the analysis of the relationship between perceived usefulness and intention to use, the empirical results show that hypothesis H7 is supported. This indicates that perceived usefulness has a significantly positive impact (*p* < 0.001) on intention to use.

According to the analysis of the relationship between social needs and intention to use, the empirical results show that hypothesis H8 is supported. This indicates that social needs have a significantly positive impact (*p* < 0.001) on intention to use. 

According to the analysis of the relationship between esteem needs and intention to use, the empirical results show that hypothesis H9 is supported. This indicates that esteem needs have a significantly positive impact (*p* < 0.001) on intention to use. 

According to the analysis of the relationship between cognitive needs and intention to use, the empirical results show that hypothesis H10 is supported. This indicates that cognitive needs have a significantly positive impact (*p* < 0.001) on intention to use. 

According to the analysis of the relationship between aesthetic needs and intention to use, the empirical results show that hypothesis H11 is supported. This indicates that aesthetic needs have a significantly positive impact (*p* < 0.001) on intention to use. 

According to the analysis of the relationship between the need for self-actualization and the intention to use, the empirical results show that hypothesis H12 is supported. This indicates that the need for self-actualization has a significantly positive impact (*p* < 0.001) on the intention to use.

Table 8 shows the difference between education level and mobile phone experience in terms of perceived usefulness, social needs, esteem needs, and self-actualization needs when analyzed using SPSS software. According to the research framework (Figure 5), the variable of path direction is assumed to be the dependent variable, and the variable of path starting is assumed to be a factor.

According to the analysis of the relationship between mobile phone experience and perceived usefulness, the actual results of this study show that hypothesis H1 is not supported. This indicates that the positive impact of mobile phone experience on perceived usefulness is not significant. 

According to the analysis of the relationship between education level and perceived usefulness, the actual results of this study show that hypothesis H2 is not supported. This means that the positive impact of education level on perceived usefulness is not significant. 

According to the analysis of the relationship between education level and social needs, the actual results of this study show that hypothesis H3 is not supported. This indicates that the positive impact of education level on social needs is not significant.

According to the analysis of the relationship between education level and esteem needs, the actual results of this study show that hypothesis H4 is supported. This indicates that education level has a significantly positive impact (*p* < 0.01) on esteem needs. 

According to the analysis of the relationship between education level and self-actualization needs, the actual results of this study show that hypothesis H5 is supported. This means that education level has a significantly positive impact on (*p* < 0.01) self-actualization needs.

### 4.4. Findings and Discussion

Based on TAM and HNT, this study constructs a demand model to confirm the needs of seniors for a healthy home-based intelligent exercise system. After analyzing the empirical data of 320 seniors using regression and difference analysis methods, this study finds that perceived usefulness (PU), perceived ease of use (PE), social needs (SNs), esteem needs (ENs), cognitive needs (CNs), aesthetic needs (ANs), and self-actualization needs (SA) positively affect the willingness of the subjects to use the home-based intelligent exercise system of golf croquet. Based on the comprehensive analysis results, the following important findings are summarized.

#### 4.4.1. The Subjects Mostly Agree with the Ease of Use of the Home-Based Intelligent Exercise System of Golf Croquet

This study indicates that the subjects are highly receptive to the home-based intelligent exercise system of golf croquet, especially in terms of perceived ease of use. The average number of each item is the highest, indicating that they believe that the system is convenient to use and that they can operate the system skillfully without the help of others.

#### 4.4.2. Aspects That Affect Intention to Use

Perceived usefulness (PU): This study notes that if a user believes that a system can increase his/her work performance or be of practical benefit to him/her, then the higher his/her willingness to use the system will be. On the contrary, the less the system helps him/her, the lower is his/her willingness to use it. The subjects recognize that the home-based intelligent exercise system of golf croquet is helpful to improve their quality of life. This will make the subjects more likely to use this system. In other words, this method has a positive effect on the limbs and waist, can strengthen muscles, and achieve exercise effects. The research findings are of great significance to the acceptance of the initial subjects. Some studies have put forward a relatively pessimistic view of the ability to predict user behavior based on subjective measurements [136,137]. The results of this study show that subjects can have a good impression of cognition when they actually communicate with each other in a group interactive way. Therefore, it is critical to ensure that the prototype of the design is fully implemented because at the initial stage, the testers expect to provide valuable insights on the acceptability of subsequently revised software/hardware products [138].

Perceived ease of use (PE): This study finds that if a user believes that a system is easy to learn, easy to use, and can operate skillfully, then the user’s willingness to use the system will be higher. On the contrary, the more complex and difficult the operation process of the system is to learn, the lower the willingness to use it. The subjects think that the home-based intelligent exercise system of golf croquet is very convenient to use without any assistance from others and can be easily used skillfully, which will make the subjects likely to use the system. In other words, this study combines robot and IoT devices, which tend to be easier to use. From the perspective of knowledge and learning [139], cognitive ease of use is based on procedural knowledge. Anderson [140] suggested that procedural learning only occurs when performing skills, such as learning by doing. This is one of the reasons why procedural learning is more gradual than declarative learning. Therefore, it reflects the ease of use related to the use of technology, which requires personal experience.

Social needs (SNs): This study presents that if a user believes that a system can increase interaction with others, then his/her willingness to use the system will be higher; otherwise, the willingness to use the system will be lower. The subjects believe that the home-based intelligent exercise system of golf croquet can increase the interaction between them and their families and help them interact with each other and accept others, which will make the subjects likely to use the system. In other words, in the mode of multi-person interaction, social relations can be enhanced. Fang [141] explored the role of interaction strategies in consumer decision-making. That study pointed out that among the diverse online communication mechanisms, some customers are hesitant to shop online given that IoT cannot provide the opportunity to inspect products before purchasing, thus increasing online interactivity of the website and the addition of product information to supplement online decision-making, which increases purchase and usage intentions. Yim et al. [142] stated that AR positively affects media and purchase intentions by generating greater novelty, immersion, interactivity, and usefulness compared with web-based product presentations.

Esteem needs (ENs): This study finds that if a system allows the user to gain confidence and self-affirmation in the process of using the system, then his/her willingness to use the system will be higher; otherwise, the willingness to use the system will be lower. The subjects believe that using the golf croquet home-based intelligent exercise system can make them feel proud, positive, confident, and respected, which will make them likely to use the system. The scoring system of golf croquet designed in this study and its ranking function can boost morale and make people feel honored. Liao et al. [143] applied the self-affirmation theory in order to examine the influence of real-world need satisfaction on online players’ loyalty. Their research results showed that users’ achievements and relationships in the real world can enhance their satisfaction with real-world needs, thereby enhancing the self-worth and loyalty of game players and further maintaining or enhancing the willingness to use.

Cognitive needs (CNs): This study notes that if users think that a system can arouse their curiosity and satisfy their thirst for knowledge during use, or if they want to enhance their use skills, then their perception of the system will increase, and the willingness to use will be higher. On the contrary, if the system cannot arouse the user’s curiosity, then the willingness to use will be lower. The subjects think that the home-based intelligent exercise system of golf croquet can make people feel curious and want to try to use it and that learning the use skills of this system is sufficient for cognitive needs. They will thus want to hone and enhance their proficiency, which will make the subjects likely to use this system. In other words, emerging technologies such as AIoT and Apps can stimulate users’ cognitive curiosity. The concept of curiosity is derived from flow theory. When people are in a state of immersion, they may be willing to interact with their environment [144]. Yoon et al. [145] investigated the influence of hedonic and utilitarian shopping values on continuous aspects that affect the intention to use online cross-border shopping. Their research results showed that hedonic value affects the continuous intention to use online shopping through the mediation of curiosity and self-efficacy.

Aesthetic needs (ANs): This study indicates that when the user thinks that a system is more recognizable in appearance, screen, and function, the higher his/her willingness to use the system will be. If the subject believes that the golf croquet home-based intelligent exercise system can be clearly identified and easy to use on the font display, and the functional design is not too complex, then the subject will likely use this system. The scoring system of golf croquet designed in this study has an elegant user interface design, which can increase the overall aesthetic feeling and thus increase the willingness to use it. Tsai et al. [146] investigated how user interface design affects the intention and attitude of the elderly to use social networking sites. Their results showed that user interface design and perceived ease of use positively relate to perceived usefulness, and an appropriate interface design will further affect adoption intention.

Self-actualization needs (SA): This study finds that if a system can make users feel a sense of achievement, excitement, and happiness in the process, then the more willing they will be to use the system. Each time the subject uses the golf croquet home-based intelligent exercise system, it is like a new challenge, which makes people want to obtain higher scores. The user process can fully exert a user’s potential, gain a sense of achievement, and feel excited and happy, which makes the subject likely to use this system. In other words, learning to use mobile phone applications can achieve self-growth for users, and they are never too old to learn. Using a survey of the basic factors of students’ perceived sense of achievement, pleasure, and willingness to learn web development, Zhang and Dang [147] found that the characteristics of teachers and teaching methods significantly affect their perceived sense of achievement, pleasure, and then their intention to learn web development.

#### 4.4.3. Dimensions Affected by Different Levels of Education

This study finds that people with different levels of education will have different views on esteem needs and self-actualization needs. The subjects with different education levels have significantly different views on the use of the home-based intelligent exercise system of golf croquet to gain pride, affirmation, confidence, and respect. Moreover, the subjects with different education levels have significantly different views on using the home-based intelligent exercise system of golf croquet to make people want to achieve higher scores and fully exert their potential, have a sense of achievement, and feel excited and happy during the use process. Maslow [25] referred to so-called self-actualized people, who are satisfied with life, can reach their potential and have creativity, and can have a loving and accepting attitude toward themselves and others. So-called esteem needs refer to all the needs required to acquire and maintain personal esteem, including the respect of others and self-respect. In a study on the level of education and job satisfaction, Solomon et al. [148] pointed out that it is theoretically inferred that education level involves a significant trade-off relationship. Because of the need for self-actualization, well-educated people will enjoy more resources from the job (including income, job autonomy, and diversity). Yu and Chang [149] explored the needs of the elderly according to the type of community. They found that seniors with high education levels have better self-confidence and value of themselves as well as their level of respect and self-actualization needs.

## 5. Conclusions

Due to economic development and the improvement in national living standards in recent years, people’s leisure and sports time has increased, their concept of physical activities has become stronger, and the time and money spent on sports have risen (Malm et al. [150]). However, the aging trend in the global population highlights the planning direction of the future leisure sports of seniors. How to choose or participate in leisure sports is thus worthy of concern and discussion. Generally speaking, old people often pursue the best quality of life, but not everyone can attain or improve to a high-quality enjoyment of life when they are old. Therefore, in the future, everyone should pay attention to the planning of lifestyle in old age. The indispensable factors are life satisfaction, self-esteem, general health and function, and social status [151,152], which can also bring proper esteem and responsibility during one’s own later years.

This study was conceived at the early stage of the COVID-19 epidemic. At that time, the problem of seniors living in elderly care centers in northern Taiwan for a long time and the lack of exercise and good social activities led to organ degradation or increased isolation and loneliness. Therefore, this research puts forward the concept of a home-based intelligent exercise system (including an intelligent scoring system, modified golf croquet, robot, and mobile phone applications) and applied for a patent design with the Republic of China at the same time. In order to understand the acceptance and demand level of seniors in this home-based intelligent exercise system, this study conducts demand analysis using TAM combined with Maslow’s needs theory so that the original leisure sports items belonging to teams and individuals can achieve the effect of daily sports, and so that seniors can also gain psychological attribution.

## 6. Contribution and Applicability of the Study

The results of this study show that the subjects have high acceptance of the home-based intelligent exercise system of golf croquet, especially in terms of perceived ease of use. This shows that they think the system is convenient to use and can operate the system skillfully without assistance from others. It is also a leisure activity with fitness and social functions, which is quite suitable for seniors. By participating in croquet, they can gain recognition from their peers and also temporarily put aside any unhappiness, depression, and anxiety, thus effectively finding a form of relief. These results are particularly interesting, as Chinese society has traditionally chosen to avoid unfamiliar information technology due to self-learning resistance or face-saving issues. Therefore, it is important to confirm the acceptance level of convenience brought by emerging technologies among Chinese people. This design framework is not only used in the golf croquet system but also widely used in the seniors’ sports management system. The application field can also be extended to the community, gym, or family, and the objects of use can also be extended to children and teenagers. Thus, these findings uniquely contribute to the intelligent exercise system literature.

Sports record Apps and fitness teaching Apps have both attracted researchers’ attention [153,154]. Current sports and fitness Apps have many problems, such as serious product homogeneity, low user viscosity, and lack of scientific innovation. However, with continuous improvement at the professional and technical level by fitness App makers, future fitness Apps should be more convenient to meet the needs of users. In addition, with optimization and improvement of user experiences using IoT, VR, or other information technologies, Apps will become more personalized for users and take into account scientific data collection and security service provision. With the cooperation and assistance of various aspects, it is believed that sports Apps can add a healthy, safe, and happy lifestyle for older generations.

## 7. Limitations

As with other works, this study also has certain limitations. We strived to be objective in data collection, dimensions, and criteria establishment, but due to the influence of external factors, there are still some unavoidable drawbacks. For example, subjective well-being is an individual’s evaluation of the subjective perception of certain things. The subjective well-being of elderly people participating in golf croquet may have other factors that affect their subjective perception and feelings. In the future, variables such as personality traits of the elderly people themselves, life satisfaction, or other factors that may affect them can be added for further research and comparison. As this study was affected by the COVID-19 epidemic, more competition activity data could not be collected due to the inability to hold more competitions, meaning any improvement in the overall design could be slightly inadequate. It was also impossible to explain the equipment and functions of this study face-to-face with the respondents, the interaction could only maintain a relative distance, and the entire process of the activity was introduced over a video. Most of the questionnaire content in this study may have been difficult or unclear for seniors to understand. In addition, most of the seniors speak Taiwanese and do not know Mandarin Chinese well, which made communication difficult and could have led to potential misunderstandings.

## Figures and Tables

**Table 1:** Literature compilation of dimension.

Dimension	Criterion	Implication	References
Perceived usefulness (PU)	Relieve loneliness	Can effectively alleviate loneliness.	[31,49]
	Stay healthy	Can maintain physical and mental health.	
Perceived ease of use (PE)	Convenience	Very convenient to use.	
	Easy to operate	Operation steps are simple.	
	Easy to learn	Does not take much time to learn.	[106,107,108]
Intention to use (IU)	Self-intention to use	Facilitate users to refer to the product and use it in practice.	
	Recommend and share	Recommend and share the product with others.	
Social needs (SNs)	Sense of identity	Can identify with each other.	[109,110]
	Maintain good relationships	Can maintain good relationships with others and maintain emotions.	
	Embrace other people	Able to accept others and live in harmony.	
Esteem needs (ENs)	Sense of glory	Can produce a sense of pride.	
	Earn recognition	Be able to gain the affirmation of others.	
	Respected by others	Be respected by others.	
	Full of confidence	Can make oneself full of confidence.	
Cognitive needs (CNs)	Curious and seek knowledge	Be curious about new sports equipment and want to know.	
	Learning their skills	Easy to learn and want to learn skills in use.	
	Hone their skills	Want to use more to improve skills.	
Aesthetic needs (ANs)	System functions	Functional design meets the requirements.	
	Font display	Font and screen display are clear and easy to recognize.	
Self-actualization needs (SA)	Exert personal potential	Be able to fully exert their potential without being disturbed by emotion or environment.	
	Competition with myself	Actively face challenges and do not give up easily.	
	Peak experience	Experience a high degree of self-realization, and can feel happy, excited, and touched in a short period of time, even selfless feelings.	

**Table 2:** Implementation schedule.

Institution\Week\Time	Tuesday/Thursday			
	13:00–14:00	14:00–15:30	15:30–16:30	Participants
Elderly care center (A)	Explanation/video introduction	Experience golf croquet	Fill out the questionnaires	192
Institution\Week\Time	Wednesday			
	13:00–14:00	14:00–15:30	15:30–16:30	Participants
Elderly care center (B)	Explanation/video introduction	Experience golf croquet	Fill out the questionnaires	128

Elderly care center (A): Every Tuesday and Thursday for a total of three weeks. Elderly care center (B): Every Wednesday for four weeks. The number of people tested every day is 32 (16 groups, 2 people in each group).

**Table 3:** Basic information of respondents.

Item	Information	Quantity	Percent
Gender	Male	263	82.2
	Female	57	17.8
Education level (EL)	Elementary school	3	0.9
	Junior high school	20	6.3
	High school	251	78.4
	University or above	46	14.4
Mobile phone experience (ME)	None	4	1.3
	Less than 1 year	0	0
	1~3 year(s)	9	2.8
	3~5 years	49	15.3
	5 years or above	258	80.6
Chronic disease	None	289	90.3
	Yes (including cardiovascular disease, diabetes, rehabilitation treatment, hypertension, cholesterol, and others)	31	9.7
Seniority of chronic disease	None	289	90.3
	Less than 1 year	3	0.9
	1~3 year(s)	8	2.5
	3~5 years	1	0.3
	5 years or above	19	5.9
Exercise times/week	0	15	4.7
	1	11	3.4
	2	39	12.2
	3	159	49.7
	4	12	3.8
	5 or above	84	26.3
Exercise duration/time	Less than 30 min	238	74.4
	1 h	62	19.4
	1~2 h(s)	15	4.7
	2~3 h	4	1.3
	3 h or above	1	0.3
Seniority of sport	Less than half year	25	7.8
	Less than year	32	10.0
	1~3 year(s)	62	19.4
	3~5 years	82	25.6
	5 years or above	119	37.2

**Table 4:** Descriptive statistics of question.

Dimension	Criterion	Question No.	Mean	SD
Perceived usefulness (PU)	Relieve loneliness	PU1	4.10	0.494
		PU2	4.00	3.92
	Staying healthy	PU3	3.97	0.461
		PU4	4.09	0.401
		PU5	4.04	0.402
Perceived ease of use (PE)	Convenience	PE1	4.20	0.461
		PE2	4.19	0.446
	Easy to operate	PE3	4.11	0.411
		PE4	4.13	0.424
	Easy to learn	PE5	4.11	0.431
		PE6	4.10	0.410
Intention to use (IU)	Self-intention to use	IU1	4.05	0.456
		IU2	4.03	0.429
		IU3	4.10	0.447
	Recommend and share	IU4	4.01	0.407
		IU5	4.01	0.375
Social needs (SNs)	Sense of identity	SN1	3.99	0.392
	Maintain good relationships	SN2	4.07	0.424
	Embrace other people	SN3	3.99	0.363
Esteem needs (ENs)	Sense of glory	EN1	3.95	0.401
	Earning recognition	EN2	3.97	0.375
	Respected by others	EN3	3.97	0.414
	Full of confidence	EN4	4.02	0.461
Cognitive needs (CNs)	Curious and seek knowledge	CN1	4.03	0.410
	Learning their skills	CN2	4.00	0.423
	Hone their skills	CN3	4.02	0.419
Aesthetic needs (ANs)	System functions	AN1	4.03	0.429
	Font display	AN2	4.03	0.399
Self-actualization needs (SA)	Exert personal potential	SA1	3.99	0.434
	Competition with myself	SA2	3.98	0.415
	Peak experience	SA3	4.00	0.419

**Table 5:** KMO measure of sampling.

	Testing	KMO	Bartlett’s Test of Sphericity		
Dimension			$\chi^{2}$	*df*	*p*
PU		0.812	511.938	10	0.000 ***
PE		0.851	2158.924	15	0.000 ***
IU		0.847	1029.735	10	0.000 ***
SN		0.722	527.225	3	0.000 ***
EN		0.789	1006.788	6	0.000 ***
CN		0.761	714.099	3	0.000 ***
AN		0.500	258.196	1	0.000 ***
SA		0.729	567.436	3	0.000 ***

PU: perceived usefulness. PE: perceived ease of use. IU: intention to use. SN: social needs. ENs: esteem needs. CN: cognitive needs. AN: aesthetic needs. SA: self-actualization needs. *** *p* < 0.001.

**Table 6:** Analysis of factor, reliability, and item-total correlation.

Question No.	Eigenvalue	Cumulative Percentage	Factor Loading	Cronbach’s α	Item-Total Correlation
PU1	2.889	57.773	0.622	0.814	0.770
PU2			0.660		0.758
PU3			0.556		0.783
PU4			0.548		0.782
PU5			0.502		0.793
PE1	4.711	78.512	0.730	0.945	0.939
PE2			0.777		0.934
PE3			0.777		0.935
PE4			0.797		0.933
PE5			0.828		0.931
PE6			0.803		0.933
IU1	3.611	72.214	0.692	0.902	0.885
IU2			0.638		0.894
IU3			0.761		0.871
IU4			0.792		0.869
IU5			0.728		0.881
SN1	2.413	80.425	0.841	0.874	0.788
SN2			0.735		0.883
SN3			0.836		0.797
EN1	3.230	80.742	0.755	0.917	0.907
EN2			0.871		0.873
EN3			0.840		0.879
EN4			0.764		0.909
CN1	2.595	86.512	0.852	0.922	0.898
CN2			0.877		0.877
CN3			0.866		0.866
AN1	1.746	87.302	0.873	0.853	0.880
AN2			0.873		0.862
SA1	2.643	82.09	0.867	0.891	0.801
SA2			0.787		0.874
SA3			0.809		0.856

Question Nos. refer to Appendix A.

**Table 7:** Regression analysis results.

Dimension	β	*T*	*p*	$R^{2}$	$Adj-R^{2}$
PU	0.700	17.464	0.000 ***	0.490	0.488
PE	0.615	13.909	0.000 ***	0.378	0.376
SN	0.793	23.174	0.000 ***	0.628	0.627
EN	0.779	22.139	0.000 ***	0.607	0.605
CN	0.770	21.545	0.000 ***	0.593	0.592
AN	0.798	17.854	0.000 ***	0.501	0.499
SA	0.742	19.738	0.000 ***	0.551	0.549

PU: perceived usefulness. PE: perceived ease of use. SN: social needs. EN: esteem needs. CN: cognitive needs. ANs: aesthetic needs. SA: self-actualization needs. *** *p* < 0.001.

**Table 8:** Analysis of variance, ANOVA.

Between Group	SS	MS	*F*	*p*
ME-PU	4.131	1.377	1.382	0.248
EL-PU	0.726	0.242	0.240	0.868
EL-SN	6.992	2.331	5.900	0.071
EL-EN	16.921	5.640	4.015	0.001 **
EL-SA	11.713	3.904	4.015	0.008 **

ME: mobile phone experience. PU: perceived usefulness. EL: education level. SN: social needs. ENs: esteem needs. SA: self-actualization needs. SS: sum of squares of deviation from mean. MS: mean-square. ** *p* < 0.01.

## Data Availability

The data that support the findings of this study are available from the corresponding author, S.-J.C., upon reasonable request.

## Appendix A

**Dimension**	**Criterion**	**Question No.**	**Questionnaire**
Perceived usefulness (PU)	Relieve loneliness	PU1	I think using the home-based exercise system of golf croquet can help me not feel lonely.
		PU2	I think that using the home-based exercise system of golf croquet can increase my interaction with others.
	Staying healthy	PU3	I think using the home-based exercise system of golf croquet can effectively achieve the effect of daily exercise.
		PU4	I think using the home-based exercise system of golf croquet can make me think about how to obtain higher scores and train brain activity.
		PU5	I think using the home-based exercise system of golf croquet can help me to properly exercise my muscles and joints and relieve the discomfort caused by chronic diseases.
Perceived ease of use (PE)	Convenience	PE1	I think the home-based exercise system of golf croquet does not need a large venue, which makes it very convenient for me to use.
		PE2	I think the home-based exercise system of golf croquet can be used without the help of many people, which makes it very convenient for me to use.
	Easy to operate	PE3	I think it is easy for me to understand the content and function of the home-based exercise system of golf croquet.
		PE4	I think the home-based exercise system of golf croquet can make it easy for me to use it alone.
	Easy to learn	PE5	I think the home-based exercise system of golf croquet can make me learn to operate it easily.
		PE6	I think the home-based exercise system of golf croquet can make me easily and skillfully operate it.
Intention to use (IU)	Self-intention to use	IU1	I would like to use the home-based exercise system of golf croquet.
		IU2	I am willing to use the home-based exercise system of golf croquet with my relatives and friends.
		IU3	If relatives and friends recommend the home-based exercise system of golf croquet to me, I will want to use it.
	Recommend and share	IU4	I will invite relatives and friends to use the home-based exercise system of golf croquet.
		IU5	I will share the home-based exercise system of golf croquet with my relatives and friends.
Social needs (SNs)	Sense of identity	SN1	I think that the home-based exercise system of golf croquet can help me gain recognition and interact with others.
	Maintain good relationships	SN2	I think the home-based exercise system of golf croquet can make me enjoy and interact with my family.
	Embrace other people	SN3	I think the process of using the home-based exercise system of golf croquet can help me accept others and win the trust of my friends.
Esteem needs (ENs)	Sense of glory	EN1	I think that using the home-based exercise system of golf croquet gives me a sense of honor.
	Earning recognition	EN2	I think the home-based exercise system of golf croquet can make me affirmed.
	Respected by others	EN3	I think using the home-based exercise system of golf croquet can make me feel respected.
	Full of confidence	EN4	I think using the home-based exercise system of golf croquet can make me full of confidence.
Cognitive needs (CNs)	Curious and seek knowledge	CN1	I think the home-based exercise system of golf croquet can make me feel novel and want to use it.
	Learning their skills	CN2	I think learning the skills of using the home-based exercise system of golf croquet can satisfy my thirst for knowledge.
	Hone their skills	CN3	I think continuous use of the home-based exercise system of golf croquet can help me increase my skills.
Aesthetic needs (ANs)	System functions	AN1	I think the function design of the home-based exercise system of golf croquet can make me willing to use it.
	Font display	AN2	I think the home-based exercise system of golf croquet can help me use the font and screen display.
Self-actualization needs (SA)	Exert personal potential	SA1	I think using the home-based exercise system of golf croquet can help me fully exert my potential and have a sense of achievement.
	Competition with myself	SA2	I think every time I use the home-based exercise system of golf croquet, it is like a new challenge, which can make me want to a higher scores.
	Peak experience	SA3	I think using the home-based exercise system of golf croquet can make me feel excited and happy.

## Appendix B

**Ethical Statement**

According to journal policy, ethical approval or exemption from the ethics committee is required before research, or authors are encouraged to cite local or national legislation that indicates that such research does not require ethical approval. This is a non-interventional study (e.g., surveys, questionnaires, social media research) where anonymity is guaranteed, and all participants are fully informed as to why the study is being conducted, how the data will be used, and whether there are any associated risks. This study does not need ethical approval according to the following Taiwan regulations:

1. Human Subjects Research Act, Chapter 2, Article 5. (https://law.moj.gov.tw/ENG/LawClass/LawAll.aspx?pcode=L0020176) (accessed on 28 September 2023).

Prior to conducting research, the principal investigator shall submit the research protocol for review and approval by the Institutional Review Board (hereinafter, IRB). However, the research protocol within the scope of exemption categories for IRB review, as announced by the competent authority, shall not apply.

2. Announcement of Ministry of Health and Welfare, Executive Yuan, Republic of China (Taiwan). Issue Date: 5 July 2012, Issue No.: 1010265075. (http://at.cdc.tw/N2G0v1) (accessed on 28 September 2023).

Scope of Human Research Cases Exempted from Ethics Approval Committee Review: The research case does not take minors, inmates, indigenous, pregnant women, people with physical and mental disabilities, mental patients, and other people who have been determined or judged by the review meeting to be under undue coercion or unable to make a decision of their own free will as research objects; and in any of the following circumstances below, they may be exempted from being sent to the ethics approval committee for review or be issued with a certificate of exemption by the ethics approval committee.

2.1 Non-anonymous, non-interactive, and non-intrusive research conducted in a public setting and in which no specific individual can be identified from the information collected.
  2.2 Use of information that has been legally made public and use of the information conforms to the purpose of its announcement.
  2.3 A study on the effectiveness evaluation of public policies conducted by public agencies themselves or entrusted by professional institutions when performing their statutory duties.
  2.4 A study of educational evaluation or testing, teaching skills, or effectiveness evaluation in a general teaching environment.
  2.5 The research project exhibits minimum risk, and the potential risk suffered by the research object is not higher than that of the researcher who did not participate in it. After evaluation by the ethics approval committee, it can be exempted from review and be issued a certificate of exemption. The minimum risk mentioned above refers to the probability or intensity of harm or discomfort suffered by the research object that is not higher than that suffered in daily life.
